# The Effect of Clinical Factors on the Reversion of Cg05575921 Methylation in Smoking Cessation

**DOI:** 10.3390/epigenomes9020012

**Published:** 2025-04-28

**Authors:** Robert Philibert, Steven R. H. Beach, Michelle R. vanDellen, James A. Mills, Jeffrey D. Long

**Affiliations:** 1 Department of Psychiatry, University of Iowa, Iowa City, IA 52246, USA; jim-mills@uiowa.edu (J.A.M.); jeffrey-long@uiowa.edu (J.D.L.); 2 Behavioral Diagnostics LLC, Coralville, IA 52241, USA; 3 Center for Family Research, University of Georgia, Athens, GA 30602, USA; srhbeach@uga.edu; 4 College of Public Health, University of Oklahoma Health Sciences, Tulsa, OK 73104, USA; michelle-vandellen@ouhsc.edu; 5 Department of Biostatistics, University of Iowa, Iowa City, IA 52242, USA

**Keywords:** cg05575921, DNA methylation, smoking cessation, contingency management, financial incentive treatments

## Abstract

Background: Financial Incentive Treatments (FIT) can be effective in the treatment of smoking. However, weaknesses in current biochemical approaches for assessing smoking cessation may hinder its implementation, particularly for management of long-term smoking cessation. The use of cg05575921 methylation assessments could address some of the shortcomings of current self-report and non-self-report methods, but additional information is needed about the speed of methylation reversion as a function of key clinical and demographic variables. Methods: To better understand those relationships, we analyzed data from 3040 subjects from the National Lung Screening Trial (NLST), including 1552 self-reported quitters. Results: Plotting of the data as a function of time since quitting shows that methylation increases approximately 14%, on average, after at least one full year of cessation with a subsequent slow non-linear increase in methylation over the next 14 years. Least Squares Regression modeling shows strong effects of quit time and a modest, yet significant, effect of body mass index (BMI) on the rate of reversion. Prior cigarette consumption characteristics and sex made modest contributions as well, with the latter largely offset by pre-cessation methylation levels. Race and age were not significant factors in the models. Conclusions: When combined with data from prior studies, these analyses of the long-term reversion of cg05575921 methylation will be informative to those considering FIT approaches to incentivizing reversion of cg05575921 as an index of short- and long-term smoking cessation.

## 1. Introduction

Smoking is the largest preventable cause of death in the world [1]. A variety of evidence-based psychological and medical therapies for smoking cessation exist [2]. But in the real world, their effectiveness has proven to be poor, particularly in those who are most heavily addicted to smoking [3]. Therefore, there is considerable interest in methods to increase their effectiveness.

One potential form of intervention—financial incentive treatment (FIT) for quitting—may be particularly useful for facilitating quit attempts. Over the past 15 years, FITs have garnered increasing amounts of attention as one method through which to increase the effectiveness of existing treatment methods. FITs provide an additional, tangible, short-term reason to quit, temporarily increasing individual motivation to follow intervention guidelines. Importantly, FITs are distinct from conceptually similar contingency management (CM) interventions. CM also targets similar health problems but uses incentives attached to highly proximal behaviors (e.g., daily abstinence). In FIT, alternative reinforcers, typically in the form of sizeable financial incentives (USD 100+), are made available contingent upon quitting or reducing smoking [4]. After concluding that FIT approaches were ethical, the United States Centers for Medicaid Services initiated a multi-state trial of FIT in smoking cessation in 2011. Overall, the result of the study supported the use of FIT for smoking cessation [5]. However, the authors noted the need for further study to examine the optimal design of incentive programs to support sustained behavior change [5,6]. Supporting that conclusion, in 2019, Notley et al. used standard Cochrane Database procedures to review the literature on 33 incentive based approaches and found *“*high certainty” that FIT based approaches increased the rate of smoking cessation regardless of the other mechanisms employed [4]. Despite using reinforcers, these interventions increase intrinsic motivation to quit, even in populations with low baseline motivations to quit smoking [7]. Thus, FITs directly address a barrier to cessation.

However, there have been at least two additional barriers to effectively applying FIT to long-term smoking cessation, both of which are related to issues in the biochemical verification of smoking status over time. The first barrier is determining who is eligible to participate in a FIT program. Because some widely used methods of biochemical verification reflect only variation in very short-term use, some participants are incentivized to “game the system”. Indeed, in our own studies of FIT, we have found that otherwise non-smoking subjects would begin smoking in the days or hours before the intake appointment in order to have positive cotinine or exhaled carbon monoxide (CO) levels [8]. Similarly, in a 2015 study by Halpern et al., cotinine testing suggested that up to 20% of the subjects who enrolled in their CM study may not have actually been long-term smokers [9]. The second barrier is difficulties in determining who has quit smoking. Because of the ubiquity of vaping and the need of many to use sustained nicotine replacement therapy to maintain abstinence, the value of cotinine testing has diminished [10]. CO testing can be employed, but the short half-life of exhaled CO can allow subjects who continue to smoke to evade detection and gain monetary reward [11]. Daily or more frequent collection of expired CO with facial recognition is possible but effortful for both participants and monitors. Therefore, for these and other reasons, there is considerable need for the development of other methods through which to determine the extent to which long-term smokers have quit and stayed quit over an extended period of time.

Development of Methylation-based assessment of smoking. In 2008, we published the first report of gene specific methylation changes in response to smoking [12]. In 2012, we published the first epigenome wide association analysis (EWAS) of DNA methylation changes in response to smoking, which identified cg05575921, a CpG residue in the aryl hydrocarbon receptor repressor (AHRR), as the most sensitive and specific locus for smoking in the methylome [13]. Since then, methylation at cg05575921 has been widely replicated as a marker of smoking intensity [14,15]. These early developments in the examination of methylation in response to smoking set the stage for applying a more sensitive and potentially clinically useful assessment method, the use of methylation sensitive digital polymerase chain (MSdPCR) assessments of cg05575921. MSdPCR is a Precision Epigenetic approach that allows a precise, reference-free assessment of methylation to be conducted in a matter of hours using standard digital PCR equipment [16,17]. Using this technique, the set point for DNA methylation in whole blood from biochemically verified non-smokers, both adult and adolescent, in the United States is approximately 87% ± 3 [16]. In response to sustained smoking (e.g., at least five cartons of cigarettes), there is a dose-dependent, non-linear demethylation of cg05575921 methylation [18]. Critically, when smoking cessation occurs, methylation at cg05575921 reverts. Short term studies have shown that changes up to 11% are observed in the first three months of cessation [8]. Conversely, after one year, a study of long terms quitters showed a non-linear reversion of cg05575921 that slowly approaches the methylation values seen in non-smokers [17].

Conceivably, if the key parameters predicting individual variability in reversion were better understood, assessments of cg05575921 methylation status could address the shortcoming of other biochemical methods and serve as a “hemoglobin A1c-like” barometer of smoking cessation status. In particular, these assessments could identify those who have successfully sustained quit attempts by examining change from initial methylation level to level assessed at follow-ups. Because the amount of re-methylation observed in the period between 3 months and 2 years varies between individuals and even average reversion curves are currently poorly constrained, additional research is needed to better constrain these curves and aid in predicting expected response to sustained cessation over time. In particular, the contribution of key clinical and demographic factors, such as sex, prior smoking intensity, and race on the degree of reversion remains unstudied. This lack of information is a barrier to the equitable implementation of this technology in CM approaches for smoking cessation.

Recently, we demonstrated a simple algorithm that uses cg05575921 status, age, and packyear consumption to accurately predict the eight-year likelihood of lung cancer in participants in National Lung Screening Trial (NLST) [19]. Interestingly, almost half of the subjects in that NLST cohort had quit smoking in the 15 years prior to study intake. In this study, we re-analyze those clinical and methylation data from those subjects to determine the degree of reversion of methylation at cg05575921 as a function of years since quitting, and the impact of demographic or smoking related clinical factors on that reversion.

## 2. Results

The relevant clinical and demographic characteristics for the 3040 subjects whose data are included in this study are given in Table 1. Subject age at study entry ranged from 55 years to 74 years of age, with an overall age of approximately 61.6 ± 4.9 years. The majority of the subjects are male (54%), with only 7% of the subjects reporting non-White race.

The cohort was nearly evenly split between self-reported current smokers (49%) and former smokers (51%). Because the study required at least 30 PYs of smoking history, only one of the subjects reported smoking 11 cigarettes per day (CPD). Both current smokers and former male smokers reported smoking two more cigarettes per day on average than female smokers (*t*-test, *p* < 0.0001). After controlling for sex, former smokers reported higher average daily cigarette consumption, while they were smoking, than current smokers (30.4 ± 11.7 vs. 25.4 ± 9.0, Least Squares, *p* < 0.0001). Similarly, after controlling for sex, former smokers tended to have a higher BMI than current smokers (Least Squares, 28.8 ± 5.1 vs. 27.0 ± 4.8, *p* < 0.0001). However, after controlling for sex, there was no difference in the number of PYs consumed between the smokers and former smokers.

As the next step of our analysis, we examined the relationship of cg05575921 methylation status to cigarette consumption and quitting status over time. Among current smokers, females tended to have higher overall levels of methylation (49.1% ± 13.7 vs. 45.8% ± 13.8, *p* < 0.0001) and both lower daily cigarette consumption (24 ± 8 vs. 26 ± 9 CPD, *p* < 0.0001) and lifetime consumption (51 ± 18 vs. 58 ± 24 PY, *p* < 0.0001) than males. After controlling for gender, there was not a significant relationship between daily cigarette consumption and cg05575921 levels in the current smokers.

**Figure 1:**
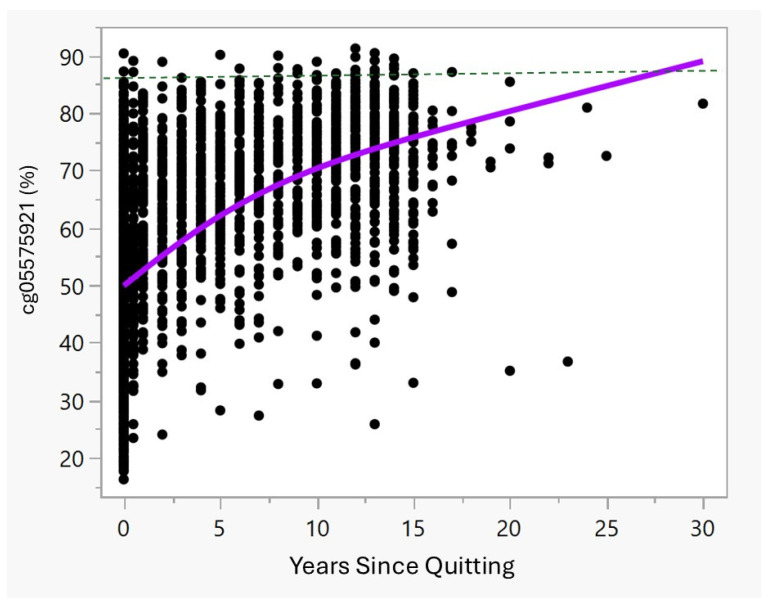
An unadjusted plot of the relationship of cg05575921 methylation to the number of years quit. The violet line is a spline fit of the reversion curve. The dashed green line is the average methylation in lifetime non-smokers in the United States.

Figure 1 and Table 2 illustrate the cg05575921 methylation and quitting status. As the data show, in the first year of quitting, the average methylation increases quickly from an average of 47.2% observed in the current smokers, with those having quit for months to up to one year having average methylation of 55.0% and those with one year since quitting having methylation of 62.5%. These values differ significantly. Then, after 2 years of cessation, methylation tends to slowly increase towards the methylation set point of 87% found in non-smokers [16].

To understand the relationship between key variables of interest to clinicians, including age, sex, PY consumption history, CPD, BMI, and race, to the reversion of DNA methylation, we conducted Least Squares Regression analysis. Because clinicians consider all aspects of the patient and both we and others have shown non-linear effects with respect to increasing time of cessation [8,17,20,21], all clinical variables were entered simultaneously and a time of cessation quadratic term was introduced. The resulting model explained approximately 21% of the variance (Adj R^2^ = 0.206, n = 1531) in the model. Table 3 provides the parameter estimates for each of the significant terms. Not surprisingly, the number of years quit had the largest impact on methylation. Sex had an impact, with males having 1.7% lower methylation as compared to females when all other variables are equal. Increasing BMI was associated with higher methylation levels (0.27% per unit, *p* < 0.0001). Interestingly, increasing PY history (−0.12% per PY, *p* < 0.0001) was associated with lower methylation while increasing daily cigarette consumption while smoking was associated with higher methylation (0.17% per cigarette, *p* < 0.0003). Since current age and race were not significantly associated with methylation, they were not included in the final regression model.

Using the parameters from the final model, we next estimated the expected values for a 55-year-old male and female subject at 1, 5, and 15 years of cessation at the 50th percentile for each of the significant variables from Table 3 for their respective sex. The average 55-year-old male with 1, 5, and 15 years of cessation would be expected to have 59.5%, 65.1%, and 70.7% methylation, respectively. The average 55-year-old female with 1, 5, and 15 years of cessation would be expected to have 61.2%, 66.8%, and 72.5% methylation, respectively.

## 3. Discussion

The potential for the use of epigenetic measures to guide clinical decision making is considerable. However, to realize that future, a precise understanding of the relationship of DNA methylation to clinical variables must be obtained so that providers can understand the potential impact of patient variability on methylation outcomes. In these analyses of data from the NLST study, we show that methylation at cg05575921 increases approximately 14% on average between year 1 and 2 after quitting, and then continues reverting upward more slowly in subsequent years as a function of time of cessation, BMI, and prior smoking characteristics. However, before further considering these data and its implications, it is important to note that data were from a cross-sectional study and the cohort was largely White and between the ages of 55 and 74. In addition, smoking cessation was assessed only by self-report. However, given that this cessation was unincentivized, there is less reason to suspect error in this self-report.

In our model of reversion of methylation level, the average male had approximately 2% lower methylation at cg05575921 at each follow-up time point than the average female. At first glance, this may suggest a different pattern of reversion for men and women. However, it should be noted that in the current smokers, the methylation in males was 3% more than in females, with males reporting smoking two cigarettes more than females each day. Since the pattern of having smoked two cigarettes per day more is also found among the former smokers, this suggests that the reason for the significant effects of sex in the model is an artifact of males being more significantly demethylated before quitting. Average reduction in males and females is very similar for a given level of cg05575921 methylation.

Similarly, at first glance, the differing slopes of the estimates for CPD and PY consumption appear confusing. But because CPD and PY are tightly correlated in the former smokers (r = 0.85), these effects largely offset one another with larger overall effects on methylation only seen at those at the extremes of the distributions for these variables (e.g., smoking 40 CPD, but with only 5 PY total consumption).

In the regression analysis, increasing BMI was significantly associated with higher levels of reversion (i.e., higher methylation). Although there are several potential explanations for this observation, one simple explanation may be that increased BMI is associated with higher levels of polyaromatic hydrocarbon (PAH) metabolism [22,23]. Cg05575921 is a CpG motif in AHRR that serves as a feedback regulator of the aryl hydrocarbon receptor (AHR) pathway [24]. Methylation at this CpG site affects the binding of a transcriptional activator to the motif in which the CpG site resides and is a measure of the level activation of the AHR, or as it is also known, the xenobiotic pathway [21,25]. Obesity is associated with both larger liver sizes and increased activity of the xenobiotic pathway, which is responsible for detoxifying the PAH found in tobacco smoke [26]. The liver is also the greatest site of overall xenobiotic pathway activity [27]. Therefore, the reason for the higher levels of cg05575921 methylation (or lower activity of the xenobiotic pathway) may be that obese people are more efficient in clearing these toxins that leach from the lungs because of their larger, more active livers and overall increased levels of xenobiotic pathway activity.

The actual amount of reversion over the first two years in heavy smokers who quit may be higher than what the current data show. In prior observations of heavily smoking subjects, we observed an 11% increase in cg05575921 methylation in just 3 months of biochemically confirmed smoking cessation. Though this is consistent with the current observations of an average of 14% after at least 1 year of cessation time point in the current study, we note considerable numbers of outlier points along the best fit methylation curve. Though speculative, we note prior reports by ourselves and others on the rate of unreliable self-report in subjects who report quitting smoking [8,9], and suspect that many, if not most, of these outliers, shown in Figure 1, are subjects who have continued to smoke, at least intermittently. If so, the rate of reversion for those with continuous smoking cessation may be considerably higher.

An unfortunate aspect of the current studies is that because many of the predictors have a strong degree of co-linearity, our ability to infer causation in some cases is limited. Conceivably, by conducting Principal Components Analyses, the degree of co-linearity could be addressed. However, that would necessitate a completely different set of analyses that would best be conducted in a longitudinal dataset with repeated assessments of key variables.

A frequent question that is asked is “how long does it take for methylation to completely revert in heavy smokers*?*” Although many subjects in this study have already achieved levels similar to those observed in non-smokers (>80%, reference), methylation in most of the quitters did not. In part, this is because the NLST study did not enroll those who had quit for more than 15 years. But in a large Japanese cohort, Takeuchi et al. followed subjects for up to three decades and used a similar MSdPCR technique to assess methylation. The overall average at cg05575921 of the group of 31 smokers (including 10 who smoked ≥ 20 PYs) who reported that they had quit for at least 30 years was 85% [17].

A major limitation of this study is that it did not include light smokers. In part, this is to be expected because the NLST was a study of the utility of LDCT screening for lung cancer. Since light smokers have a much lower rate of lung cancer than heavy smokers, they were not included as a matter of efficiently powering the study. However, we will note that the Prostate, Lung, Colorectal, and Ovarian Cancer Screening Trial has large numbers of lighter smokers, including many with non-White ancestry [28]. We are hopeful that future examinations of this or similar collections will serve additional insight into the rate of reversion in lighter smokers and to more rigorously examine potential effects of ethnicity on the rate of reversion.

What are the implications of this study for the use of cg05575921 methylation to inform smoking cessation? First, in our opinion, these data suggest that those with low BMI may revert more slowly than others. Given the small parameter estimates listed in Table 3, this effect on the overall degree of methylation (e.g., 14% between Year 1 and 2) is likely to be minimal, except for those at the extremes of BMI. As discussed above, sex did have a significant effect in the models listed in Table 3. But this is likely offset by the higher average methylation found in female smokers before they stopped smoking. Although the number of non-White subjects was limited in this study, race does not seem to have an effect, and we note the prior results by Takeuchi et al. showing very similar results in a large Japanese cohort [17]. Still, if methylation is to be used clinically, prudence dictates a need for further examination for effects of race not evident in this study, as well as for effects across the lifecycle. There is an additional need for ongoing studies to better understand the reversion process across the lifecycle and in all patients. For example, the effect of medications, such as the herbal supplement berberine, which induces AHR activity, will need to be tabulated. In addition, since the current approaches focus on assessments of white blood cells, the impact of hematopoietic disorders will also need to be understood.

When these data are considered with prior data, they provide a strong framework for initial implementation of methylation-based assessment of smoking status in FIT, and potentially even incentivization of methylation reversion itself. The data suggest BMI has only a minor impact on the rate of reversion, although it clearly should be assessed in research utilizing methylation approaches to verify abstinence. Considering this, with knowledge of an individual’s cigarette smoking at time of cessation, reversion can be reasonably predicted for a given individual. The reversion rate suggests methylation reversion is well-suited to capture cessation of one year. Finally, it should be emphasized that MSdPCR assessments are just one tool through which to assess smoking cessation. Best Practices FIT approaches may need to rely on a combination of assessments across cotinine, expired CO, and methylation change to increase the rate of sustained smoking cessation. Indeed, a focus on expired CO may be warranted in the short-term while the methylation changes accrue over time.

In summary, we describe the long-term reversion of cg05575921 methylation in response to smoking cessation. Although BMI has a nominal impact on reversion, the largest effects are carried by sustained abstinence of at least one year. These data will be useful to those considering the use of methylation-based FIT approaches for smoking cessation.

## 4. Materials and Methods

The clinical and methylation data used in this study are from the NLST study and have been previously described [29,30,31]. The NLST enrolled 53,454 US smokers, adults ages 55 to 74 with a 30-pack-year (PY) smoking history who were either current smokers or had quit within the previous 15 years at 33 sites across the United States. Smokers with less than 30 PY smoking history, having an age outside the age range, or being unwilling to be randomized were excluded from the study. The subjects were then randomized to receive either low dose computed tomography (LDCT) or chest X-ray screening arms, then annually examined each year for the occurrence of lung cancer. As part of that study, DNA was collected from 10,233 of those subjects. The DNA and data used in this study are from 3040 subjects who participated in the LDCT arm of the study and whose methylation was determined previously [19].

The clinical data were provided by the National Cancer Institute Cancer Data Access System (CDAS). Aliquots of DNA were provided by the Eastern Cooperative Oncology Group-American College of Radiology Imaging Network (ECOG-ACRIN; https://ecog-acrin.org/). The procedures used to analyze data and determine DNA methylation were approved by the NCI National Clinical Trials Network Core Correlative Sciences Committee. The number of years quit was determined by subtracting the current age to the stated age of quitting. If the resultant was “0” (i.e., less than one calendar year), the number was recoded to 0.5 for the sake of plotting and to be consistent with the data that have been produced by the Prostate, Lung, Colorectal, and Ovarian Cancer Screening Trial [32]. Body mass index (BMI) was calculated by dividing weight in pounds by the square of the subject height in inches, then multiplying by 705.

Assessments of cg05575921 methylation were conducted as previously described using methylation sensitive digital polymerase chain reaction (MSdPCR) [19]. In brief, nested primer, fluorescent probe sets from Behavioral Diagnostics, and digital PCR reagents/machinery from Bio-Rad (Carlsbad, CA, USA) were used to amplify aliquots of DNA that were bisulfite converted using Epitect Fast 96 DNA Bisulfite Conversion kits (Qiagen, Germantown, MD, USA). After amplification, the number of droplets containing amplicons with at least one “C” allele (methylated CpG residue), one “T” allele (unmethylated CpG residue), or neither allele was then determined using Bio-Rad QX-200 droplet reader. Then, the percent methylation was calculated by fitting the observed ratios of C, T, or CT containing droplet to a Poisson distribution by the proprietary Bio-Rad software (QuantaSoft Version 1.7.4).

Data analysis: DNA methylation status for 3123 subjects from the original NLST cohort supplied to us as previously described was successfully obtained. Secondary to discrepancies in some of the clinical data from these subjects (30 subjects who stated that they were still smoking but provided a quitting age, and 53 subjects who stated they had quit smoking but did not provide a quitting age), data from 83 subjects were excluded, leaving an effective cohort size of 3040.

Between group comparisons of continuous variables were compared using Student’s *T*-Test. Because of the potential confounding by sex effects, least squares regression of the effects of key variables such as BMI were conducted controlling for sex [33]. In order to understand the importance of clinical variables on the rate of reversion of methylation, multivariable least squares regression was conducted, as implemented in JMP Version 18 (SAS Institute, Cary, NC, USA) [34].

## Figures and Tables

**Table 1:** Clinical and Demographic Characteristics of NLST Subjects.

	Males	Females
N	1786	1254
Age	61.8 ± 5.0	61.2 ± 4.8
Ethnicity		
White	1656	1185
African American	75	46
Asian	22	9
Native American	5	6
Other	28	8
Hispanic	42	14
Cigs per Day (CPD) Distribution in Current Smokers		
1–10	1	-
11–20	442	419
21–30	232	158
31–40	127	62
41–60	32	14
61–80	1	-
Cg05575921 Methylation in each CPD Bin in Current Smokers		
1–10	-	-
11–20	47.1% ± 13.5	49.6% ± 14.0
21–30	44.5% ± 13.6	47.5% ± 12.9
31–40	43.0% ± 14.5	47.1% ± 13.5
41–60	46.6% ± 13.5	51.5% ± 19.0
61–80	-	-
Current Smoker Averages (n)	835	653
Cg05575921 methylation	46% ± 14	49% ± 14
Cigs per day	26 ± 9	24 ± 8
PY Consumption	58 ± 24	51 ± 18
BMI	27.3 ± 4.3	26.5 ± 5.3
Former Smoker Averages (n)	951	601
Years quit of ex-smokers	7.5± 5.1	6.9 ± 4.7
Cg05575921 methylation	66% ± 12	69% ± 11
Cigs per day while smoking	32 ± 12	30 ± 11
PY Consumption	59 ± 25	52 ± 21

**Table 2:** Clinical and Demographic Characteristics of Year 1 and 2 Quitters.

	Males	Females
Quit 1 Year	63	49
Quit 2 Years	63	49
Age	61.8 ± 5.0	61.2 ± 4.8
Ethnicity		
White	121	94
African Am.	1	3
Asian	1	-
Native Am	2	
Other	1	1
BMI	28.8 ± 5.4	28.6 ± 5.3
Cg05575921 methylation		
Year 1 Quitter	62% ± 12	63% ± 12
Year 2 Quitter	59% ± 11	65% ± 11
Overall	61% ± 11	64% ± 12
Cigs per day	30 ± 11	26 ± 11
PY Consumption	64 ± 27	55 ± 24

**Table 3:** Least Square Regression of Methylation Reversion.

	Estimate	Std Error	t Ratio	Prob > |t|
Intercept	56.560455	1.657775	34.12	<0.0001
Sex (male)	−1.732366	0.282506	−6.13	<0.0001
BMI	0.2668342	0.053153	5.02	<0.0001
CPD	0.1636593	0.047025	3.48	<0.0005
PY Consumption	−0.116133	0.022699	−5.12	<0.0001
Years Quit	0.8818391	0.062487	14.11	<0.0001
(Years Quit-7.32)^2^	−0.061402	0.010229	−6.00	<0.0001

## Data Availability

The methylation data for this study are being deposited with the NCI CDAS database in accordance with NOT-OD-19-121 guidelines.

## Abbreviations

The following abbreviations are used in this manuscript:

NLST	National Lung Screening Trial
BMI	Body mass index
FIT	Financial Incentive Treatments
CM	Contingency Management
CO	Carbon monoxide
MSdPCR	methylation sensitive digital polymerase chain
LDCT	low dose computed tomography
NCI	National Cancer Institute
CDAS	Cancer Data Access System
ECOG	Eastern Cooperative Oncology Group
ACRIN	American College of Radiology Imaging Network
CPD	Cigarettes per day
PY	Pack year
PAH	polyaromatic hydrocarbon
AHR	aryl hydrocarbon receptor
AHRR	aryl hydrocarbon receptor repressor
CpG	Cytosine-phospho-guanine
